# Supplementary material for: Structures of the Escherichia coli type 1 pilus during pilus rod assembly and after assembly termination
Source: Nat Commun. 2025 May 29;16:4988. doi: 10.1038/s41467-025-60325-z (PMC12122975; doi:10.1038/s41467-025-60325-z)
Supplement: Supplementary file 1 — Supplementary Information [file 41467_2025_60325_MOESM1_ESM.pdf]

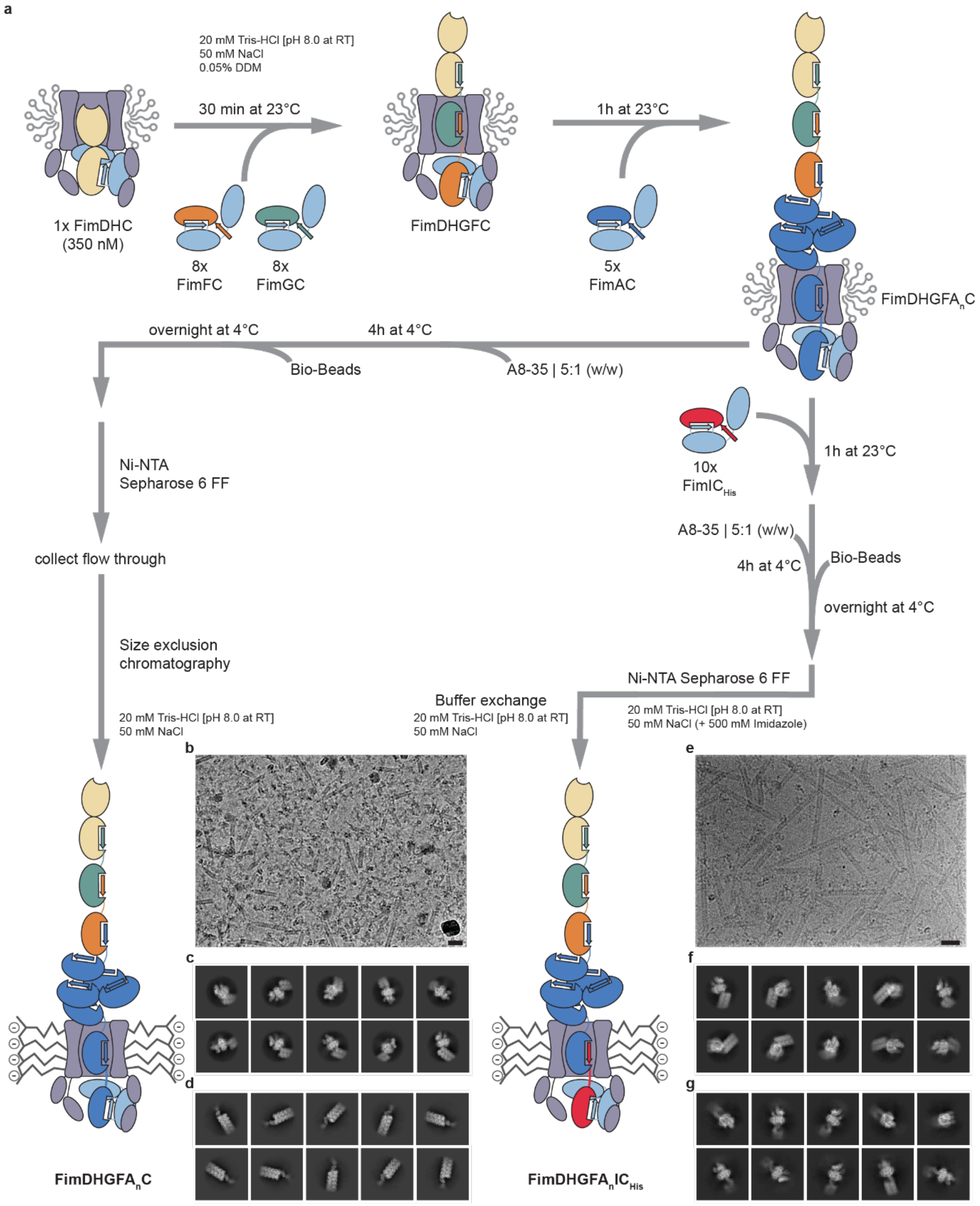

Supplementary Figure 1 | *In vitro* assembly reaction scheme, exemplary micrographs and representative 2D class averages of the FimDHGFA$_n$C and FimDHGFA$_n$IC$_{His}$ complexes. **a)** Schematic representation of *in vitro* assembly reaction and purification of FimDHGFA$_n$C and FimDHGFA$_n$IC$_{His}$ complexes before sample preparation for cryo-EM. **b)** A low-pass filtered micrograph from the FimDHGFA$_n$C complex dataset collected at 130,000 x magnification. Scale bar equals 200 Å. **c)** Selected, representative 2D classes of the FimA-bound assembly platform of the complex in (b). **d)** Selected, representative 2D class averages of the tip-to-rod transition of the complex in (b). **e)** A low-pass filtered example micrograph from the FimDHGFA$_n$IC$_{His}$ complex dataset collected at 165,000 x magnification. Scale bar equals 200 Å. **f)** Selected 2D classes of the FimDHGFA$_n$IC$_{His}$ complex. **g)** Selected 2D class averages of the FimDHGFA$_n$I$_2$C$_{His}$ complex.

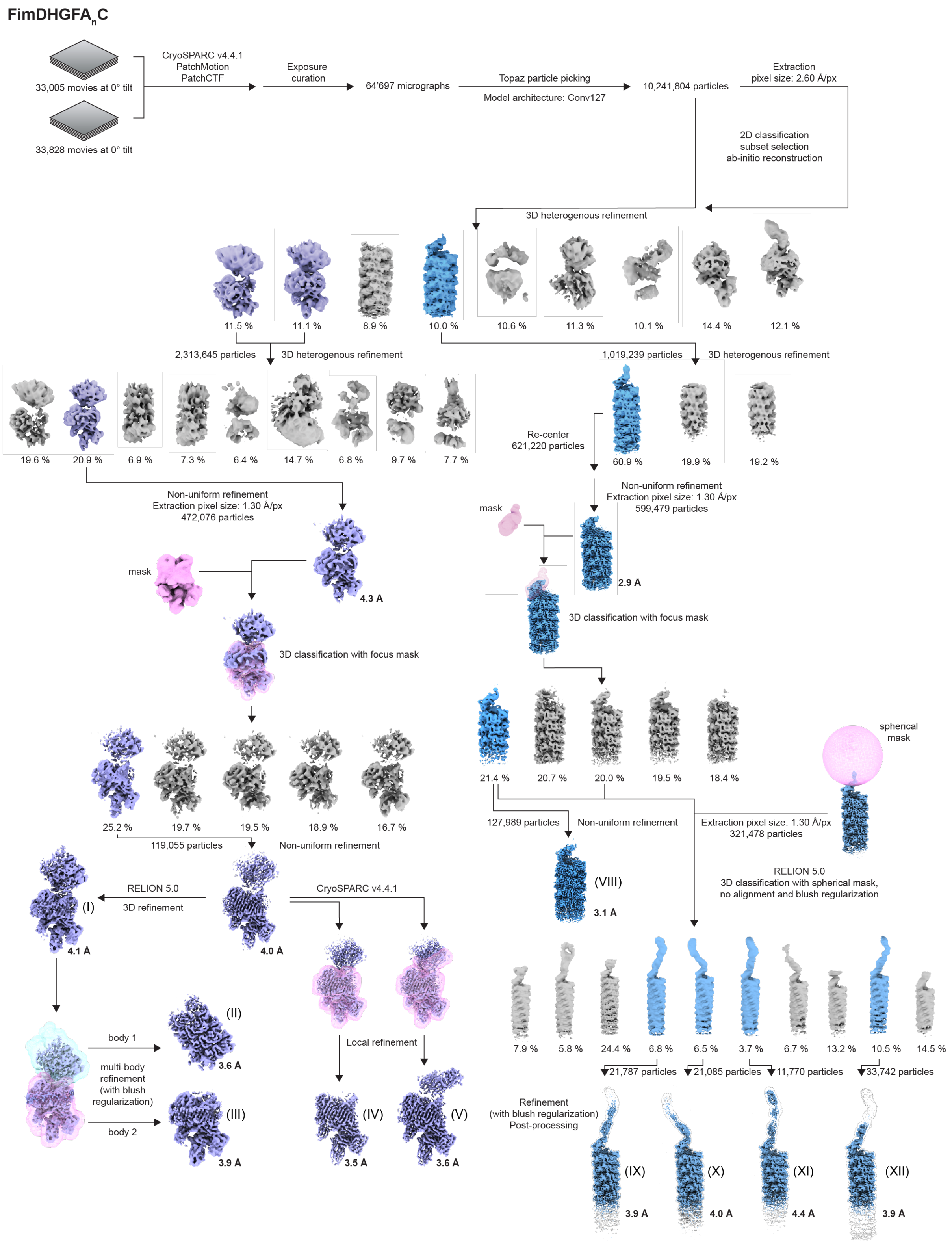

Supplementary Figure 2 | Cryo-EM data processing workflow of FimDHGFA$_n$C complex using CryoSPARC v4.4.1 and RELION-5.0 software packages. Details about the data processing workflow are described in the Methods section. Structures of the FimA-bound assembly platform (purple) and the tip-to-rod transition (blue) were solved from the same datasets. Cryo-EM maps which were further analyzed are numbered I-XII.

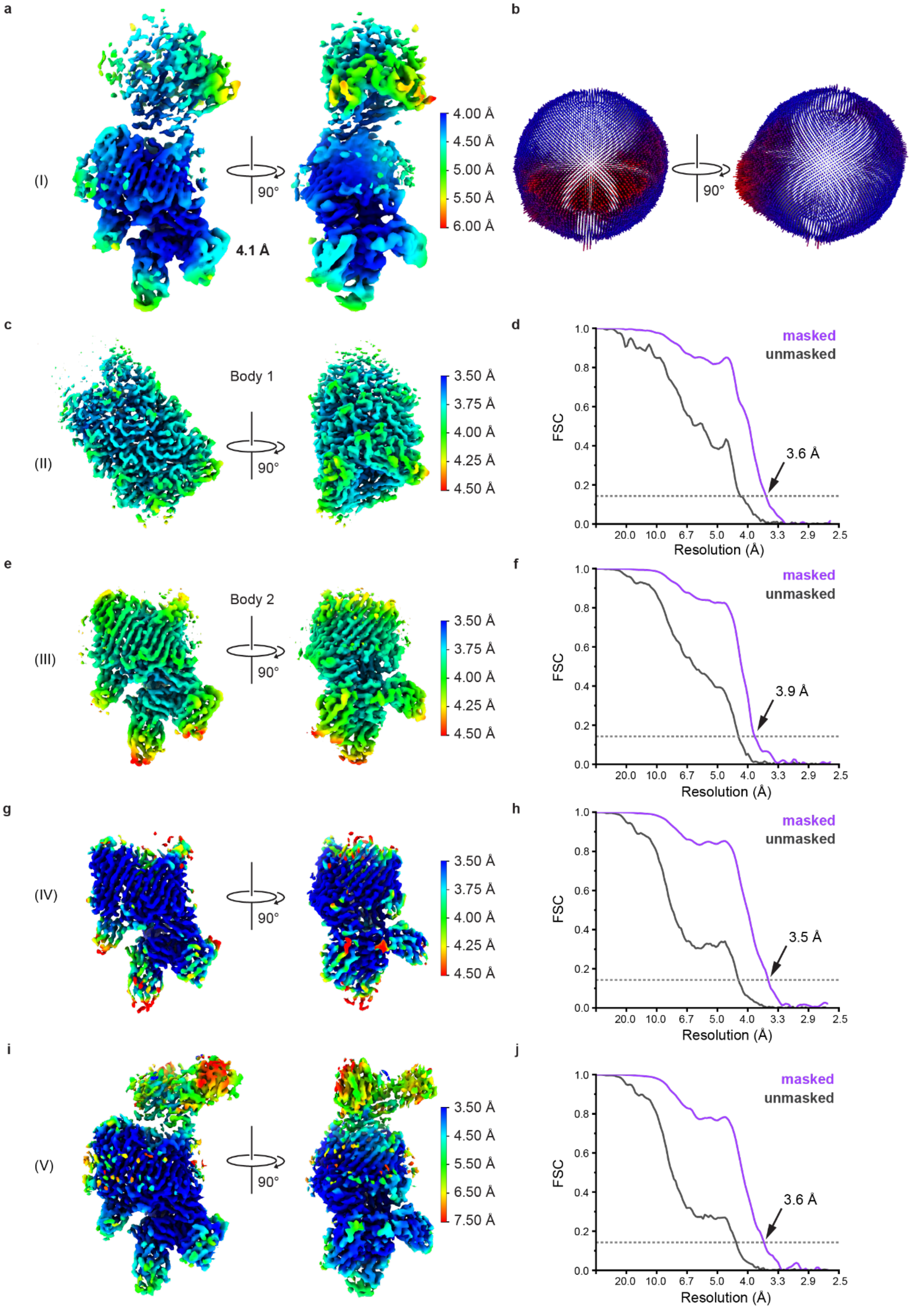

Supplementary Figure 3 | Distribution of Euler angles, local resolution plots and validation of FimDHGFA$_n$C complex maps. **a)** Consensus map of FimDHGFA$_n$C complex (map I) colored according to local resolution calculated with RELION. **b)** Angular distribution of particles corresponding to map I. **c)** Coulomb potential map of body 1 (map II) from multi-body refinement

colored according to local resolution calculated in RELION. **d)** Corresponding Fourier shell correlation (FSC) plot of map II. FSC cutoff at 0.143 is shown as a dashed line and the corresponding resolution value is indicated. **e)** Map of body 2 (map III) from multi-body refinement colored according to local resolution calculated in RELION. **f)** Corresponding FSC plot of map III. **g)** Map of assembly platform after local refinement (map IV) colored according to local resolution calculated in CryoSPARC. **h)** Corresponding FSC plot of map IV. **i)** Map of assembly platform and first FimA subunits of the pilus rod after local refinement (map V) colored according to local resolution calculated in CryoSPARC. **j)** Corresponding FSC plot of map V.

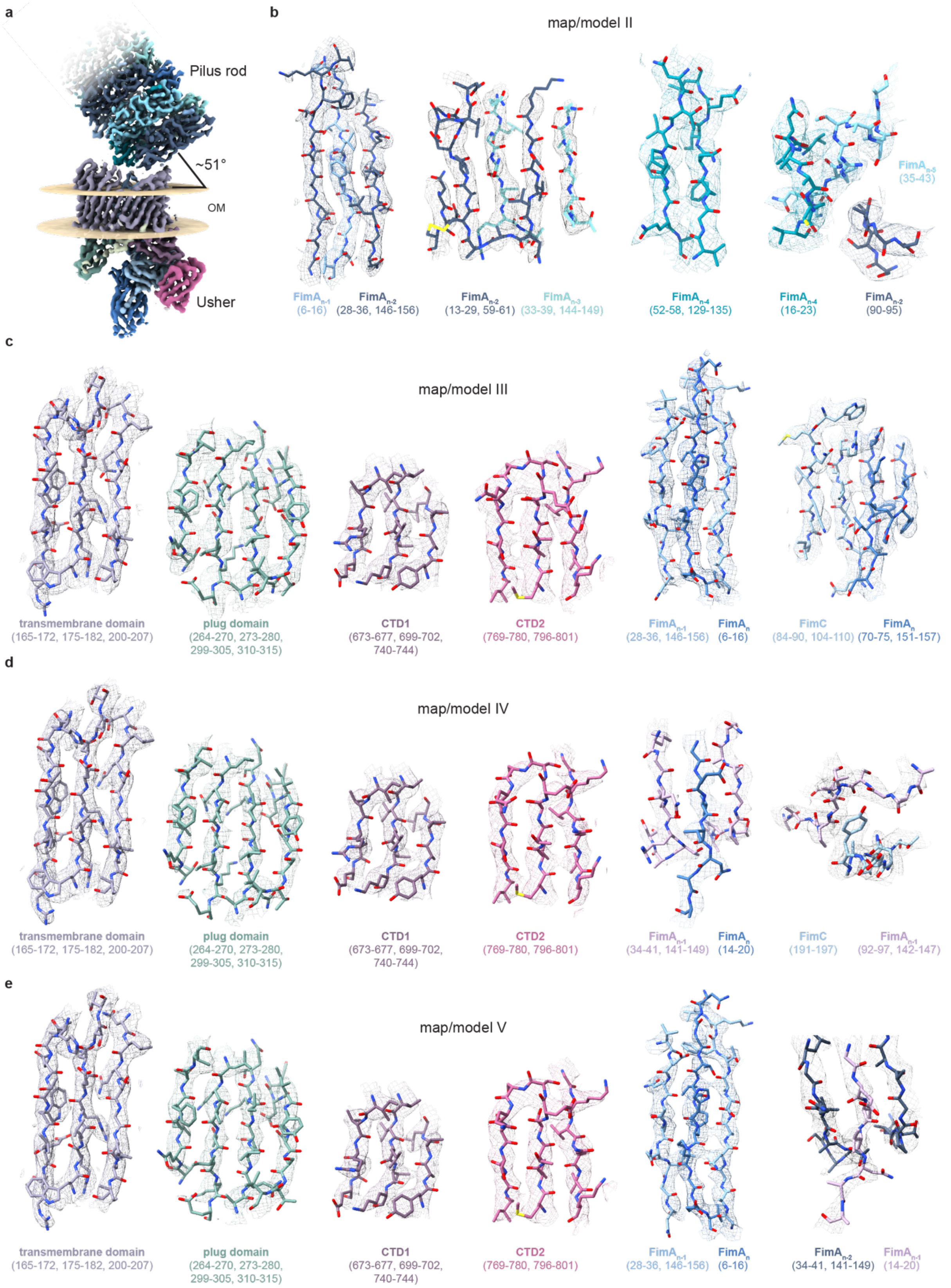

Supplementary Figure 4 | Orientation of the pilus rod with respect to the outer membrane and example densities of FimDHGFA$_n$C complex maps. **a)** Composite cryo-EM map of FimDHGFA$_n$C (map II and III) is shown with membrane boundaries of the outer membrane calculated using the PPM 3.0 web server [1]. The angle between the central axis of the pilus rod and the plane of the outer boundary was determined using ChimeraX. **b)** Cryo-EM density of map II and corresponding model for several FimA subunits. **c)** Cryo-EM density of map III and corresponding model for the different domains of FimD, FimA$_{n-1}$,

$FimA_n$ and FimC. **d)** Cryo-EM density of map IV and corresponding model for the different domains of FimD, $FimA_{n-1}$, $FimA_n$ and FimC. **e)** Cryo-EM density of map V and corresponding model for the different domains of FimD, $FimA_{n-2}$, $FimA_{n-1}$, $FimA_n$ and FimC.

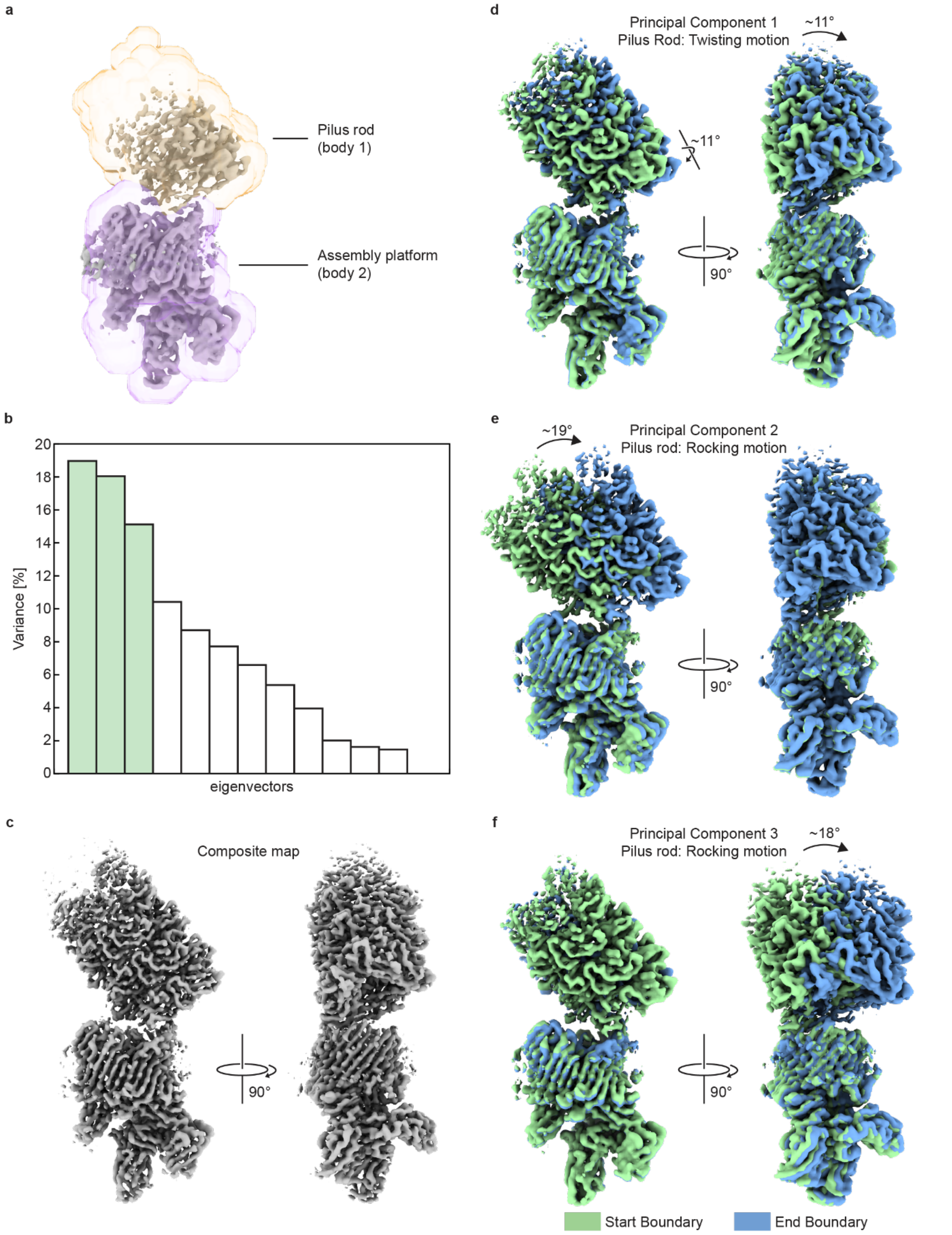

Supplementary Figure 5 | Flexibility analysis of the pilus rod by multi-body refinement reveals distinct motions in the FimDHGFA$_n$C complex. a) Masks used to define bodies for multi-body refinement. The mask surrounding the pilus rod (body 1) is colored orange while the mask for the assembly platform (body 2) is colored rose. **b)** Eigenvectors and their contribution to variance. The first three eigenvectors are colored pink. Their corresponding motions are displayed in panels d) – f). **c)** Composite map of the two bodies from multi-body refinement. **d)** Principal component 1 shows twisting motion around

the pilus rod axis. **e)** Principal component 2 shows left to right rocking motion. **f)** Principal component 3 shows front to back rocking motion of the pilus rod.

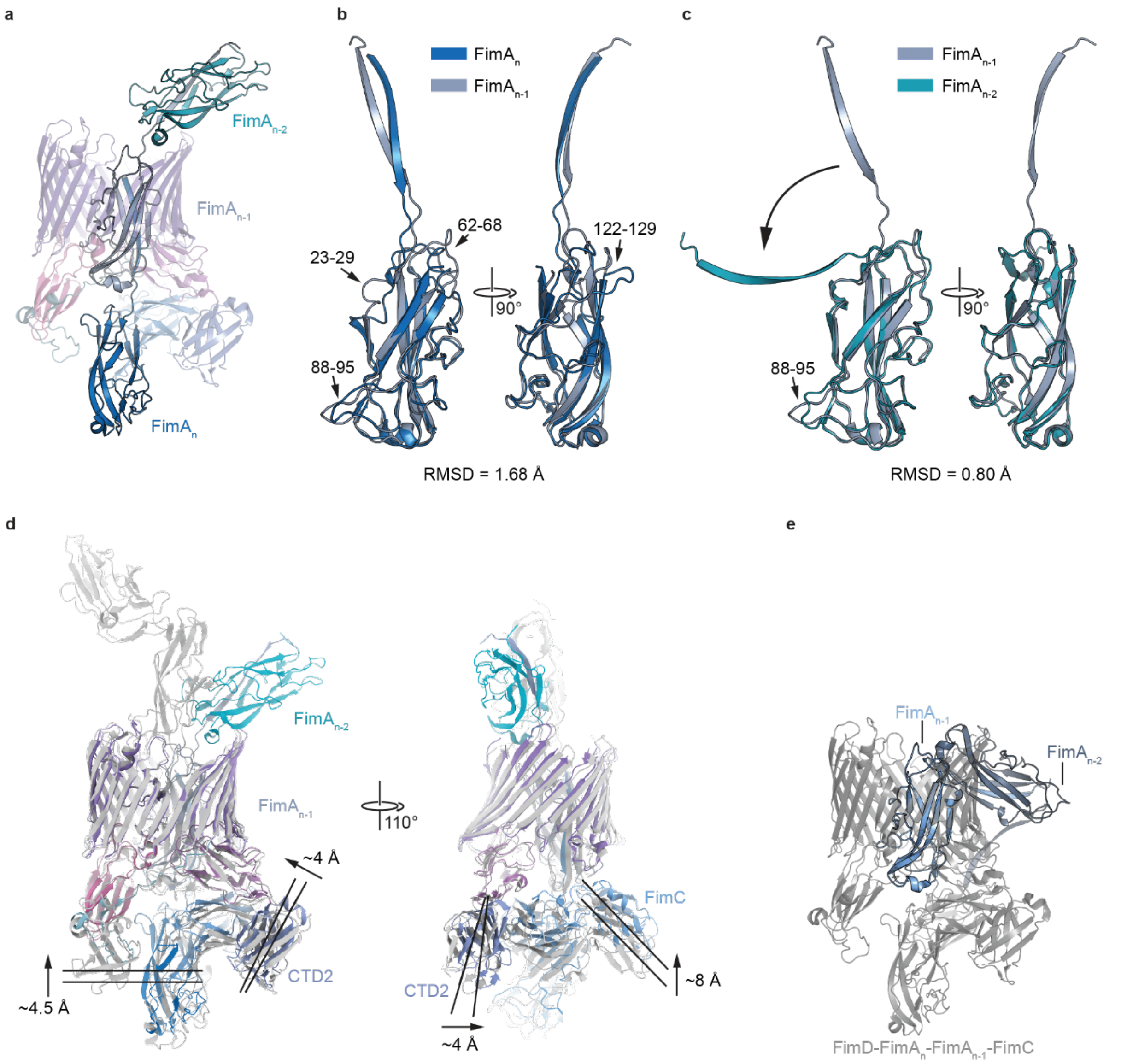

Supplementary Figure 6 | Conformational change of FimA during pilus assembly and comparison of FimDHGFA$_n$C complex to FimD-tip complex. a) Cartoon representation of model V (Local refinement of FimD-FimA$_n$-FimA$_{n-1}$-FimA$_{n-2}$-FimC). **b)** Alignment of residues 20-159 of FimA$_n$ and FimA$_{n-1}$ from the model in (a). Small conformational changes are observed in loops 23-29, 62-68, 88-95, and 122-129 as indicated by arrows. **c)** Alignment of residues 20-159 of FimA$_{n-1}$ and FimA$_{n-2}$ from the model in (a). Almost no conformational changes within the pilin domain of FimA are observed between these two positions besides loop residues 88-95. The Nte undergoes a major change in orientation from an almost linear arrangement in FimA$_{n-1}$ to the steep angle found in the pilus rod in FimA$_{n-2}$. **d)** Comparison of the FimA-bound model in (a) with conformer 2 of the FimDHGFC complex (PDB-ID 6E15 [2]). The two models were aligned based on the transmembrane domain of FimD. The overall orientation of the periplasmic domains of FimD is similar. The plug domain sits in the same position, as well as the NTD which is free and flexible. The subunit incorporated last, FimA is sitting slightly higher compared to FimF in 6E15. The CTD2 is also slightly moved resulting in FimC being moved upwards. **e)** Alignment based on residues 20-159 of a FimA subunit of the pilus rod (map II) with subunit FimA$_{n-1}$ of the model in (a). FimD-FimA$_n$-FimA$_{n-1}$-FimC of the model in (a) are colored grey, while the aligned rod subunit and its preceding subunit FimA$_{n-2}$ are colored in light blue and dark blue, respectively. If the angle between two subunits found in the pilus rod was adopted between subunits FimA$_{n-1}$ and FimA$_{n-2}$, FimA$_{n-2}$ would sterically clash with the transmembrane domain of FimD.

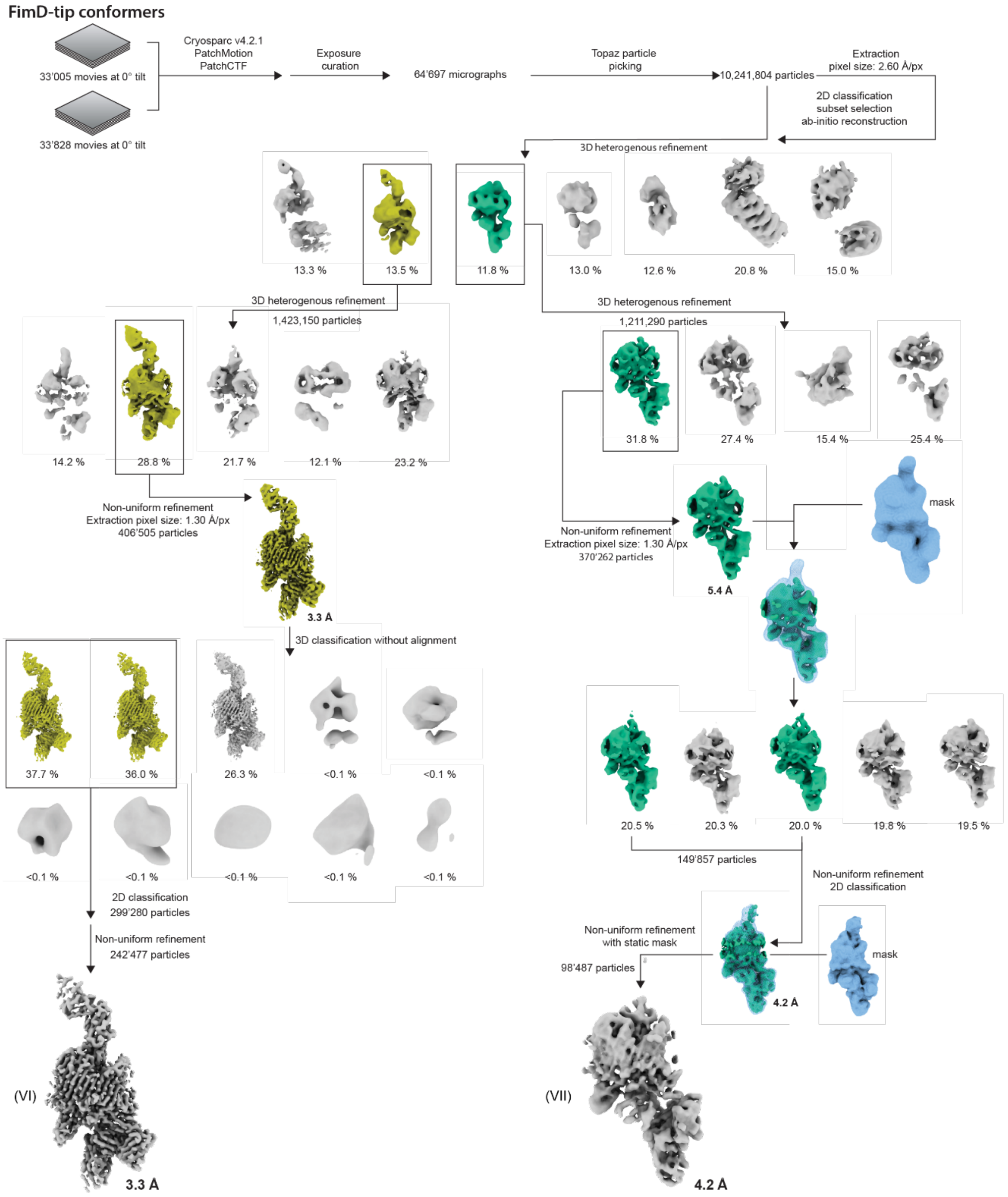

Supplementary Figure 7 | Schematic overview of the cryo-EM data processing workflow of FimD-tip complexes. Only cryoSPARC was used for processing this data. The workflow on the left leading to a structure of the FimD-tip complex with a conformation highly similar to PDB-ID 6E15 is highlighted in yellow (FimD-tip conformer 1, map VI). The workflow on the right (green) gives a 3D reconstruction of a FimD-tip complex in which both the NTD and CTDs are bound to the last incorporated chaperone-pilin complex (handover conformation), but the resolution is not sufficient to identify the subunits with confidence (FimD-tip conformer 2, map VII).

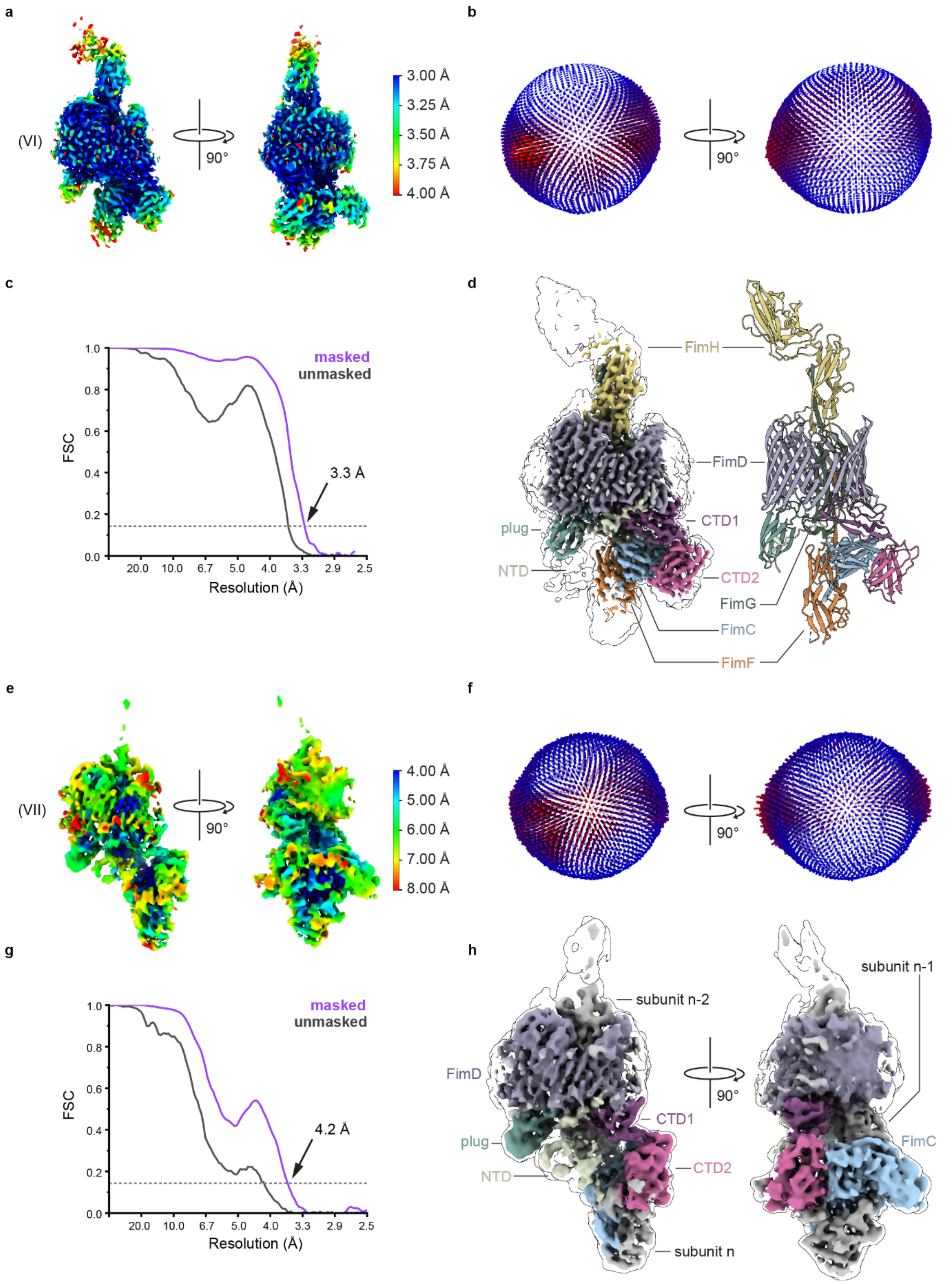

Supplementary Figure 8 | Overview of FimD-tip conformers 1 and 2. a) Cryo-EM map VI of FimD-tip conformer 1 colored according to local resolution calculated in cryoSPARC. **b)** Orientational distribution of particles corresponding to map VI displayed in (a). **c)** FSC plot corresponding to map VI in (a). **d)** Overview of the FimD-tip conformer 1 (FimDHGFC). Cryo-EM map (left) is colored according to the corresponding model VI (right). The unsharpened map is shown in the background. This conformation is highly similar to PDB-ID 6E15 [2]. **e)** Cryo-EM map VII of FimD-tip conformer 2 colored based on local

resolution calculated in cryoSPARC. **f)** Orientational distribution of particles corresponding to map VII in (e). **g)** FSC plot corresponding to map VII in (e). **h)** Cryo-EM map of FimD-tip conformer 2 colored based on models of FimD and FimC. Domains of FimD and FimC were individually rigid-body fitted in UCSF Chimera before coloring. The three subunits sitting above FimD, in the pore and on the periplasmic side, respectively, could not be unambiguously assigned and are colored in gray.

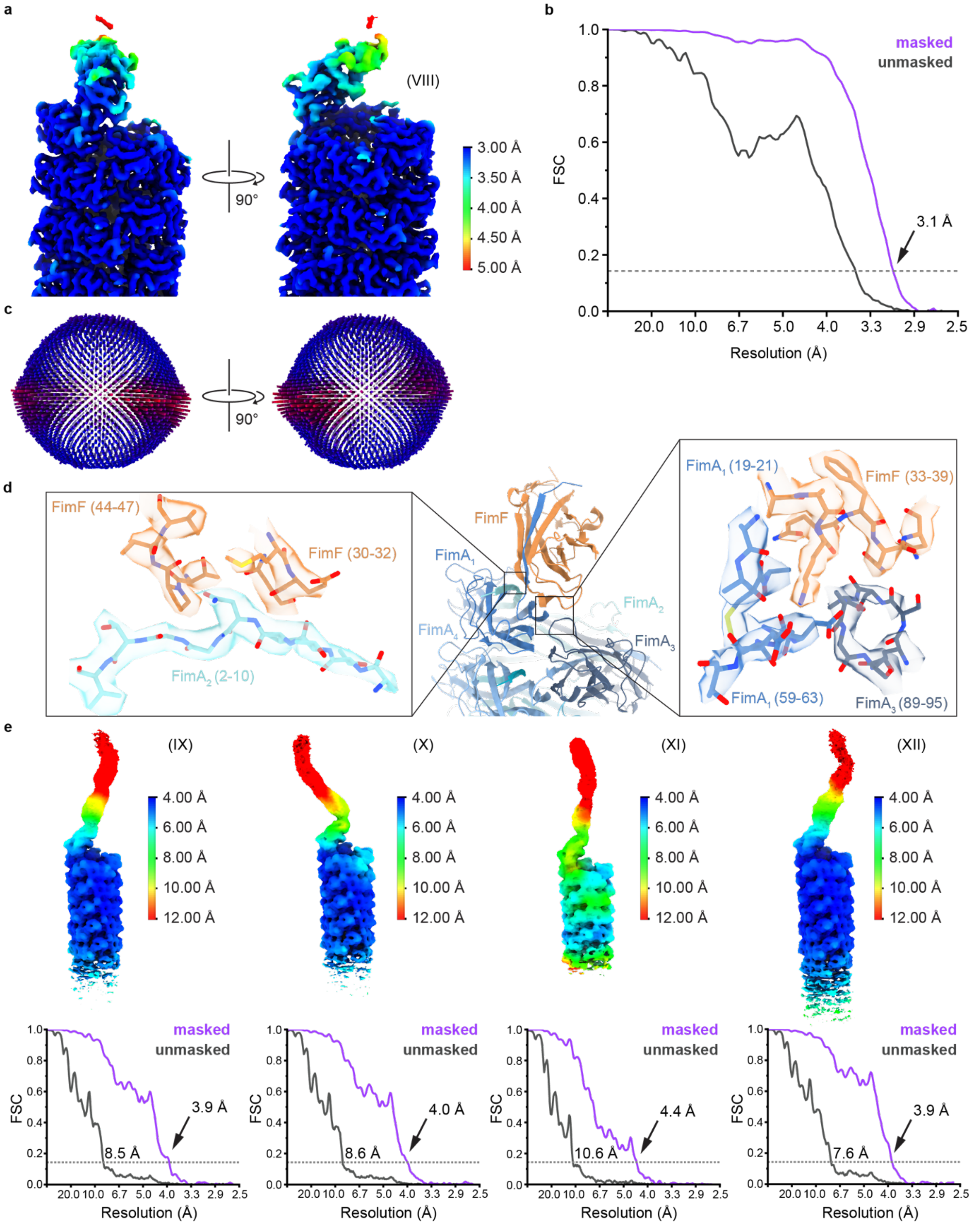

Supplementary Figure 9 | Local resolution, FSC curve, angular distribution, and cryo-EM density of the type 1 pilus tip-to-rod transition. a) Coulomb potential map colored based on local resolution calculated in cryoSPARC. **b)** FSC curve of independent half maps. The FSC threshold of 0.143 used to determine the map resolution is indicated by a dashed line. **c)** Orientational distribution of particles after non-uniform refinement in cryoSPARC. Particles are mostly distributed around the pilus rod axis. **d)** Example of Cryo-EM densities colored according to pilus subunit and corresponding atomic model. Cartoon representation of the atomic model is shown in the center. Left panel shows densities at surface between FimF (orange) and $FimA_2$ subunit (blue green). Right panel shows densities at surface of FimF, $FimA_1$ (blue) and $FimA_3$ (dark blue).

e) Cryo-EM maps of tip fibrillum classes (maps IX-XII) colored based on local resolution calculated using RELION-5.0. Corresponding FSC plots are shown below each map. FSC cut-off of 0.143 is shown as a dashed line.

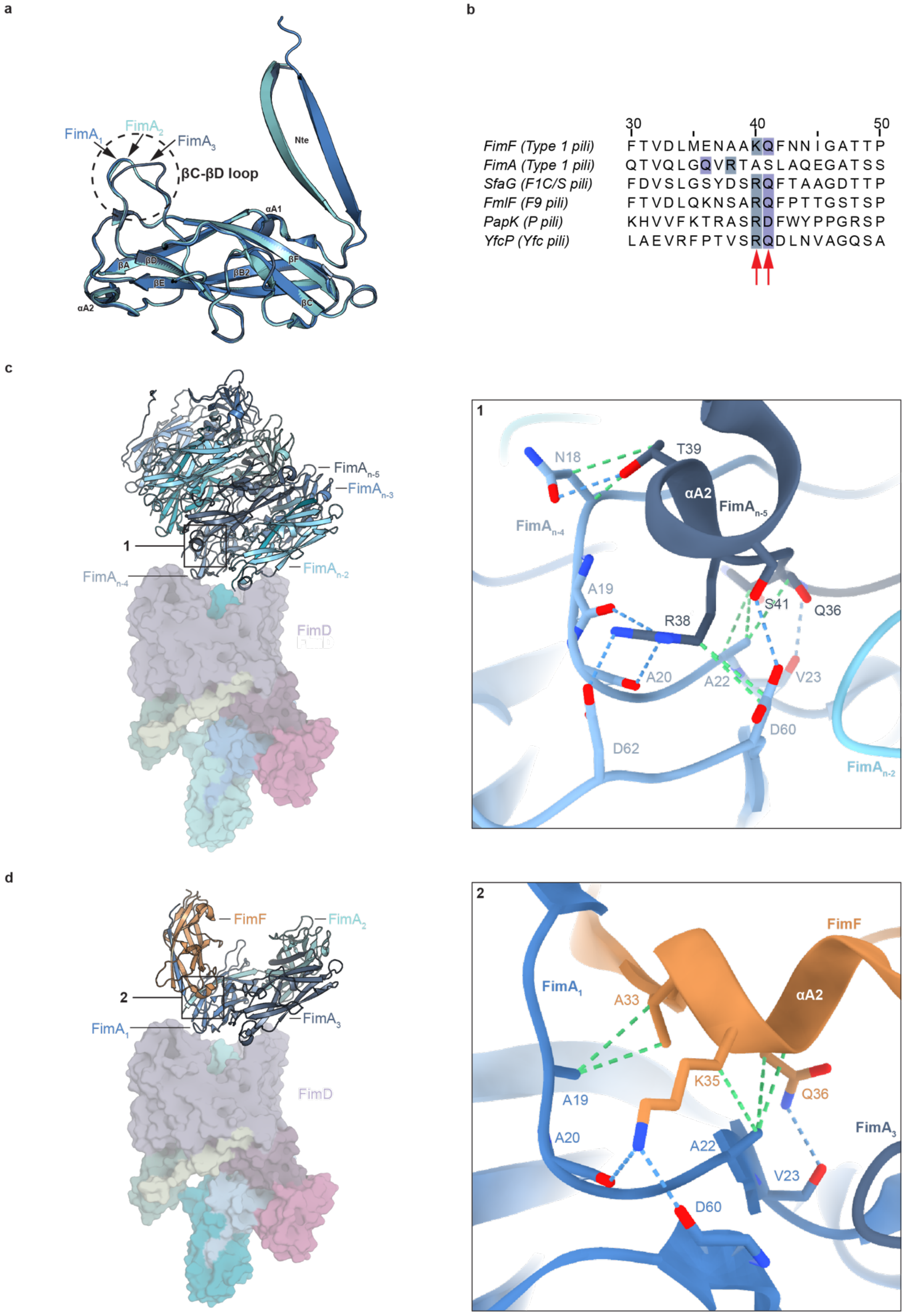

Supplementary Figure 10 | The role of FimF during formation of the first turn of the pilus rod. a) Conformational change of βC-βD loop of FimA upon interaction with FimF. While the βC-βD loop of $FimA_1$ and $FimA_2$ does not interact with any other subunit, the same loop in $FimA_3$ interacts with FimF and its conformation changes as soon as the first turn of the pilus rod is

closed. Subunits $FimA_1$ (middle blue), $FimA_2$ (blue green) and $FimA_3$ (dark blue) of the tip-to-rod transition model built into cryo-EM map VIII were superimposed in PyMOL based on residues 20-160 **b)** Multiple sequence alignment of FimF (Type 1 pilus) with FimA (type 1 pilus) and tip adaptor subunits SfaG (F1C/S pilus), FmlF (F9 pilus), PapK (P pilus) and YfcP (Yfc pilus). Conserved residues important for interaction of the tip adaptor subunit with the preceding pilus rod subunit are indicated with a red arrow. Key residues involved in interaction with the succeeding subunit are highlighted in violet and dark turquoise. Protein sequences were aligned using ClustalΩ [3] as part of the MPI Bioinformatics Toolkit [4, 5]. **c)** Overview of the composite model of the $FimDHGFA_nC$ complex built into cryo-EM maps II and III. The pilus rod is displayed as a cartoon representation while the usher with the last two incorporated FimA subunits is displayed as a surface. Panel 1 shows details of the interaction of residues in the αA2 loop of $FimA_{n-5}$ (dark blue) with the succeeding subunit $FimA_{n-4}$ (light blue). Hydrogen bonds are shown as blue dashed lines, hydrophobic interactions as green dashed lines. **d**) Structural model generated by superimposing the tip-to-rod transition model based on the first three rod subunits (FimA1, FimA2, FimA3) with the first three subunits exiting the pore ($FimA_{n-2}$, $FimA_{n-3}$, $FimA_{n-4}$) in (c). FimF and the first three FimA subunits are displayed as a cartoon representation while the usher with the last two incorporated FimA subunits is displayed as a surface. The model shows how FimF could contribute to stabilizing the first turn of pilus rod assembly by interacting with $FimA_1$ and $FimA_3$. Panel 2 shows details of interaction between residues of the αA2 loop of FimF with the succeeding subunit $FimA_1$. Hydrogen bonds are indicated by blue dashed lines while hydrophobic interactions are shown as green dashed lines.

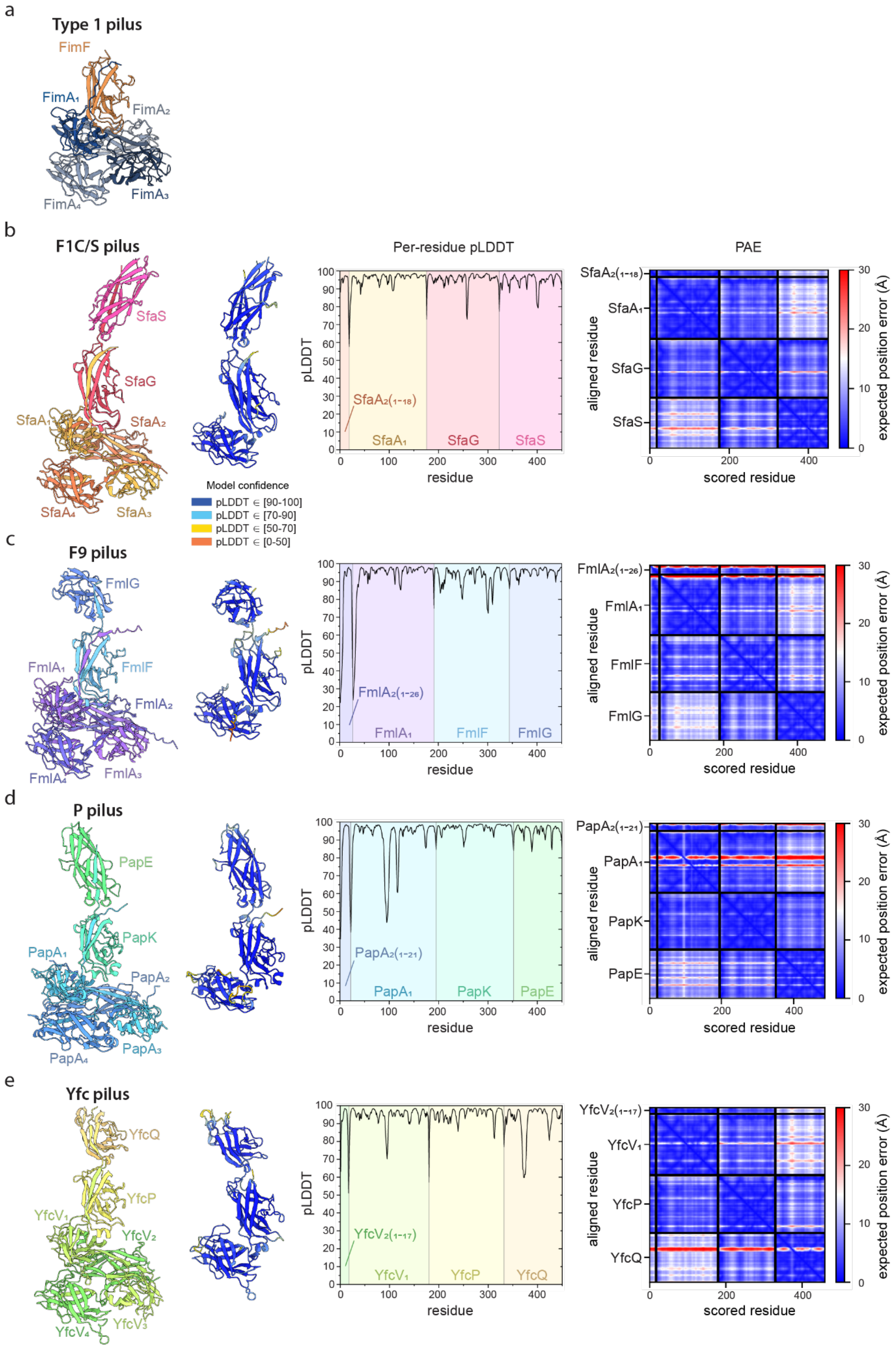
a
Type 1 pilus
FimF
FimA₁
FimA₂
FimA₃
FimA₄
b
F1C/S pilus
SfaS
SfaG
SfaA₁
SfaA₂
SfaA₃
SfaA₄
Model confidence
pLDDT ∈ [90-100]
pLDDT ∈ [70-90]
pLDDT ∈ [50-70]
pLDDT ∈ [0-50]
Per-residue pLDDT
pLDDT
residue
SfaA₂(1-18)
SfaA₁
SfaG
SfaS
PAE
aligned residue
scored residue
expected position error (Å)
c
F9 pilus
FmlG
FmlF
FmlA₁
FmlA₂
FmlA₃
FmlA₄
FmlA₂(1-26)
d
P pilus
PapE
PapK
PapA₁
PapA₂
PapA₃
PapA₄
PapA₂(1-21)
e
Yfc pilus
YfcQ
YfcP
YfcV₁
YfcV₂
YfcV₃
YfcV₄
YfcV₂(1-17)

Supplementary Figure 11 | Comparison of experimentally determined Type 1 pilus tip structure to alphafold predictions of chaperone-usher pilus tips with similar operon organization. a) Cartoon representation of Type 1 pilus tip-to-rod transition. **b**) Cartoon representation of composite Alphafold model of F1C/S pilus tip-to-rod transition (left). Alphafold prediction of two tip subunits (SfaS and SfaG) and one rod subunit ($SfaA_1$) complemented by the donor strand of the next subunit ($SfaA_{2(1-18)}$) is shown colored according to pLDDT values (middle left). Corresponding plot of per-residue pLDDT values against residue number (middle right). Corresponding PAE plot of prediction (right). **c**) Cartoon representation of composite Alphafold model of F9 pilus tip-to-rod transition (left). Alphafold prediction of two tip subunits (FmlG and FmlF) and one rod subunit ($FmlA_1$) complemented by the donor strand of the next subunit ($FmlA_{2(1-26)}$) is shown colored according to pLDDT values (middle left). Corresponding plot of per-residue pLDDT values against residue number (middle right). Corresponding PAE plot of prediction (right). **d**) Cartoon representation of composite Alphafold model of P pilus tip-to-rod transition (left). Alphafold prediction of two tip subunits (PapE and PapK) and one rod subunit ($PapA_1$) complemented by the donor strand of the next subunit ($PapA_{2(1-21)}$) is shown colored according to pLDDT values (middle left). Corresponding plot of per-residue pLDDT values against residue number (middle right). Corresponding PAE plot of prediction (right). **e**) Cartoon representation of composite Alphafold model of Yfc pilus tip-to-rod transition (left). Alphafold prediction of two tip subunits (YfcQ and YfcP) and one rod subunit ($YfcV_1$) complemented by the donor strand of the next subunit ($YfcV_{2(1-17)}$) is shown colored according to pLDDT values (middle left). Corresponding plot of per-residue pLDDT values against residue number (middle right). Corresponding PAE plot of prediction (right).

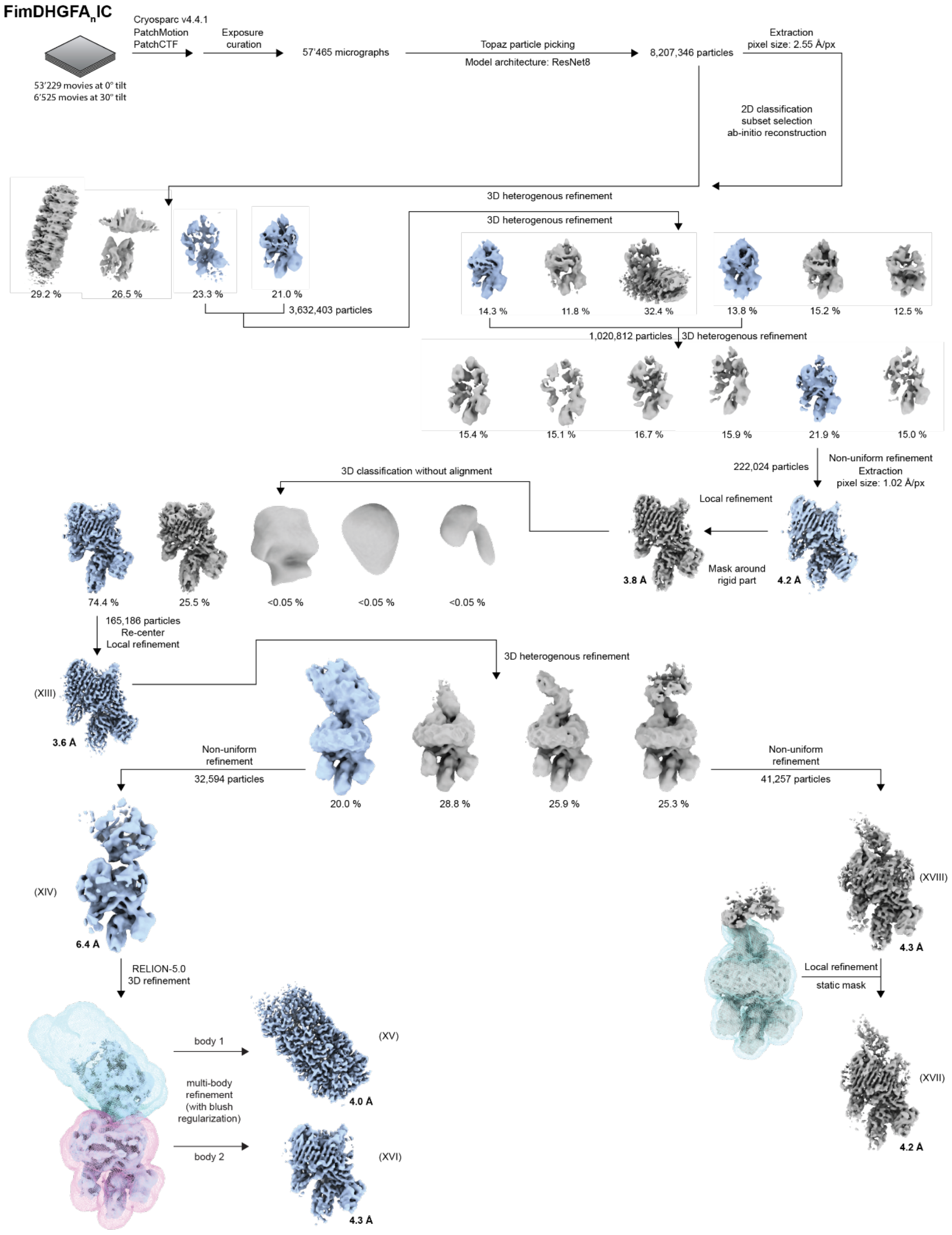

Supplementary Figure 12 | Schematic overview of the cryo-EM data processing workflow for the FimDHGFA$_n$IC$_{His}$ and FimDHGFA$_n$I$_2$C$_{His}$ complexes. Details about data processing are described in the Methods section. Cryo-EM maps which were further analyzed are numbered XIII-XVIII.

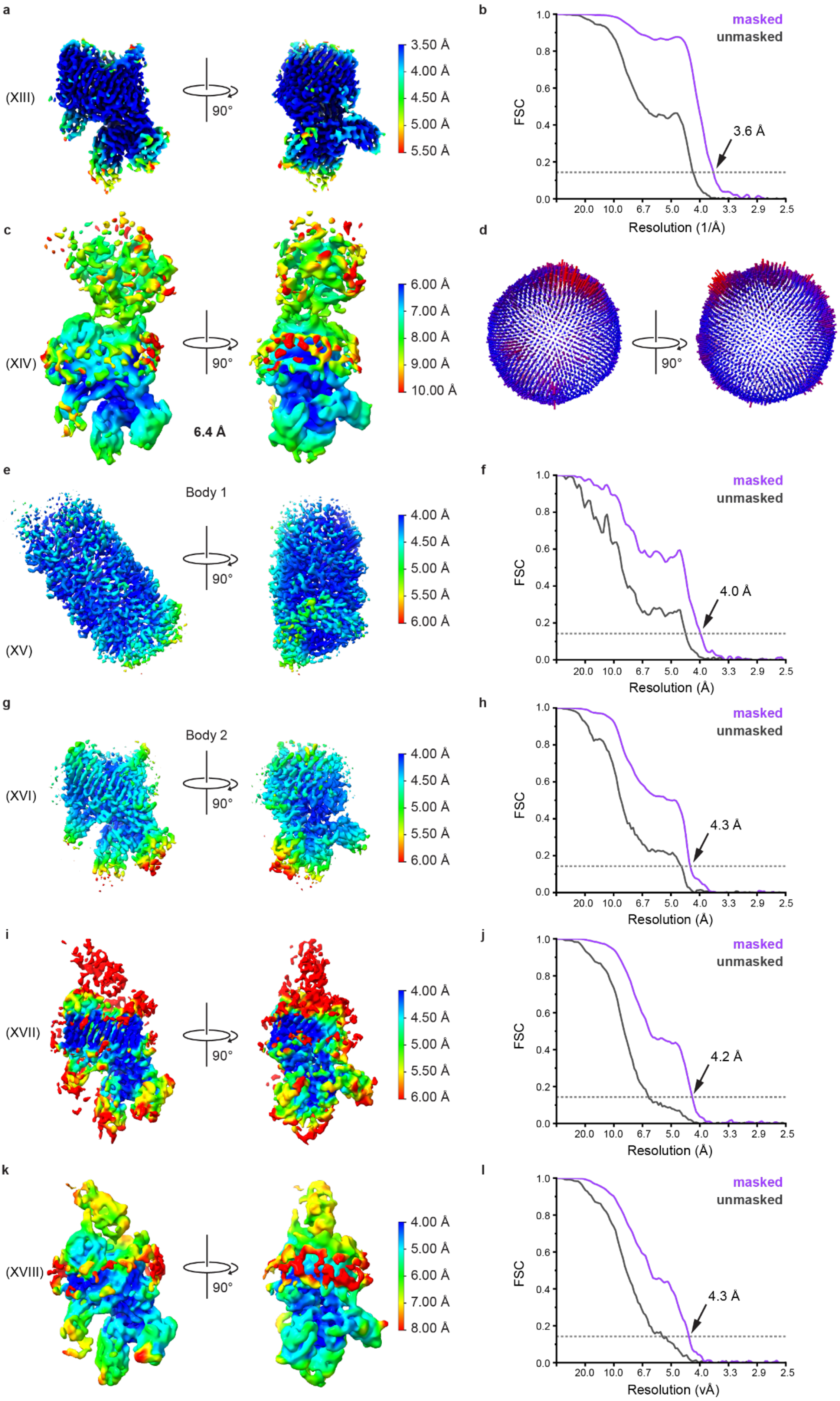
a
(XIII)
90°
3.50 Å
4.00 Å
4.50 Å
5.00 Å
5.50 Å
b
masked
unmasked
FSC
3.6 Å
Resolution (1/Å)
c
(XIV)
90°
6.4 Å
6.00 Å
7.00 Å
8.00 Å
9.00 Å
10.00 Å
d
90°
e
Body 1
(XV)
90°
4.00 Å
4.50 Å
5.00 Å
5.50 Å
6.00 Å
f
masked
unmasked
FSC
4.0 Å
Resolution (Å)
g
Body 2
(XVI)
90°
4.00 Å
4.50 Å
5.00 Å
5.50 Å
6.00 Å
h
masked
unmasked
FSC
4.3 Å
Resolution (Å)
i
(XVII)
90°
4.00 Å
4.50 Å
5.00 Å
5.50 Å
6.00 Å
j
masked
unmasked
FSC
4.2 Å
Resolution (Å)
k
(XVIII)
90°
4.00 Å
5.00 Å
6.00 Å
7.00 Å
8.00 Å
l
masked
unmasked
FSC
4.3 Å
Resolution (vÅ)

Supplementary Figure 13 | Local resolution plots, validation and distribution of Euler angles for FimDHGFA$_n$IC$_{His}$ and FimDHGFA$_n$I$_2$C$_{His}$ complex maps. a) Initial cryo-EM map of the FimI-bound usher (XIII) colored according to local resolution calculated in cryoSPARC. **b)** FSC plot corresponding to map (XIII). **c)** Consensus refinement map of the FimDHGFA$_n$IC$_{His}$ complex (map XIV) colored according to local resolution calculated in cryoSPARC. **d)** Corresponding FSC plot to map XIV in (c). **e)** Map of body 1 (map XV) from multi-body refinement of the FimDHGFA$_n$IC$_{His}$ complex colored according to local resolution calculated in RELION. **f)** FSC plot corresponding to map XV in (e). g) Cryo-EM map of body 2 (map XVI) from multi-body refinement of the FimDHGFA$_n$IC$_{His}$ complex colored according to local resolution calculated in RELION. **h**) Corresponding FSC plot to map XVI in (g). **i)** Cryo-EM map of the FimDHGFA$_n$I$_2$C$_{His}$ complex (map XVII) from local refinement colored according to local resolution in cryoSPARC. **j)** Corresponding FSC plot to map XVII in (i). **k)** Consensus refinement cryo-EM map of the FimDHGFA$_n$I$_2$C$_{His}$ complex colored according to local resolution calculated in cryoSPARC. **l)** Corresponding FSC plot map XVIII in (k).

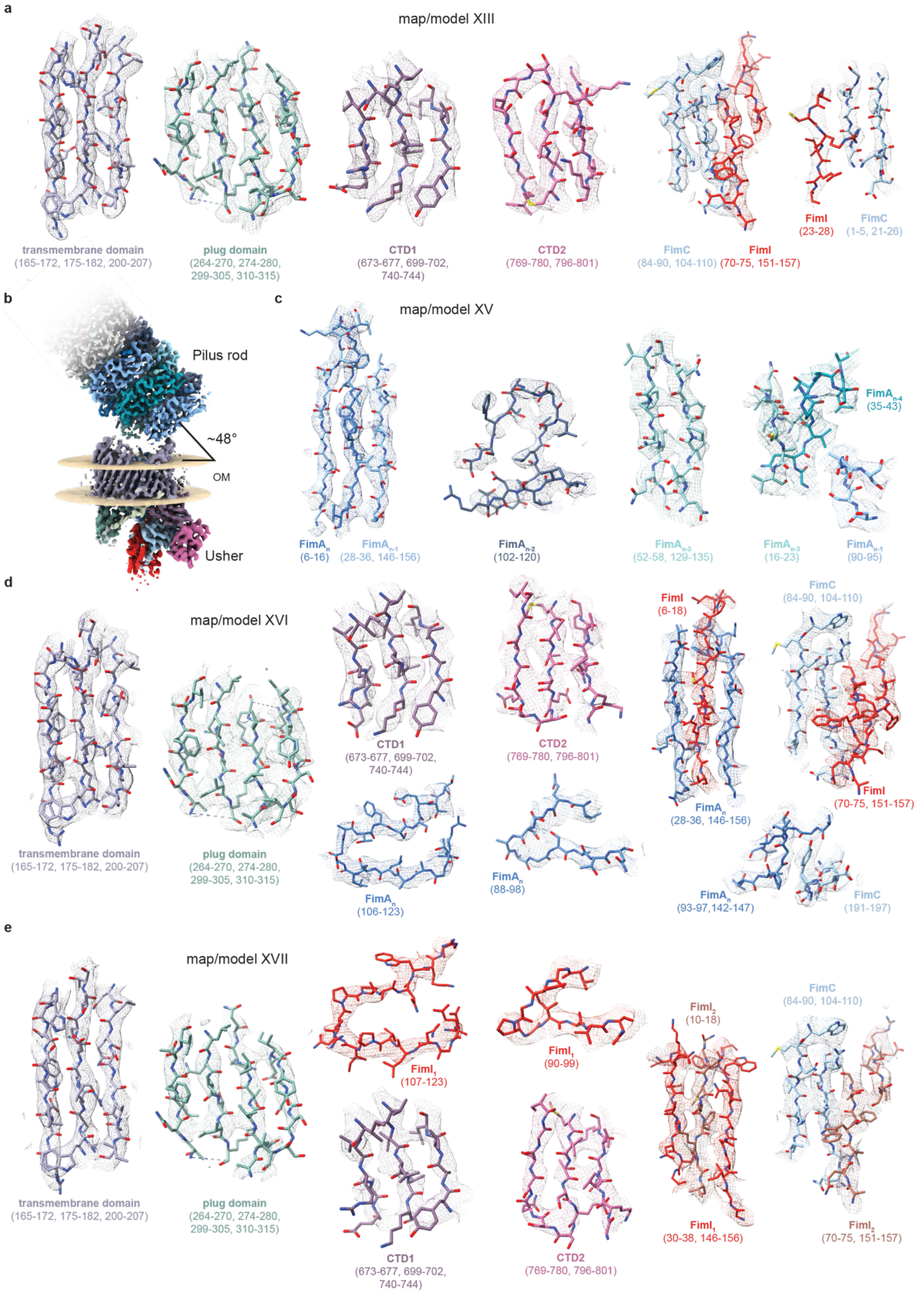

Supplementary Figure 14 | Example densities of cryo-EM maps and models of FimI-bound complexes. a) Example densities of map XIII and corresponding model for the different domains of FimD, FimI and FimC. b) Composite cryo-EM map of FimDHGFA$_n$IC$_{His}$ (map XV and XVI) is shown with membrane boundaries of the outer membrane calculated using the PPM 3.0 web server [1]. The angle between the central axis of the pilus rod and the plane of the outer boundary was determined using

ChimeraX. **b)** Cryo-EM density of map XV and corresponding model for different FimA subunits within the pilus rod. **c)** Cryo-EM density of map XVI and corresponding model for the different domains of FimD, $FimA_n$, FimI and FimC. **d)** Cryo-EM density of map XVII and corresponding model for the different domains of FimD, $FimI_1$, $FimI_2$ and FimC.

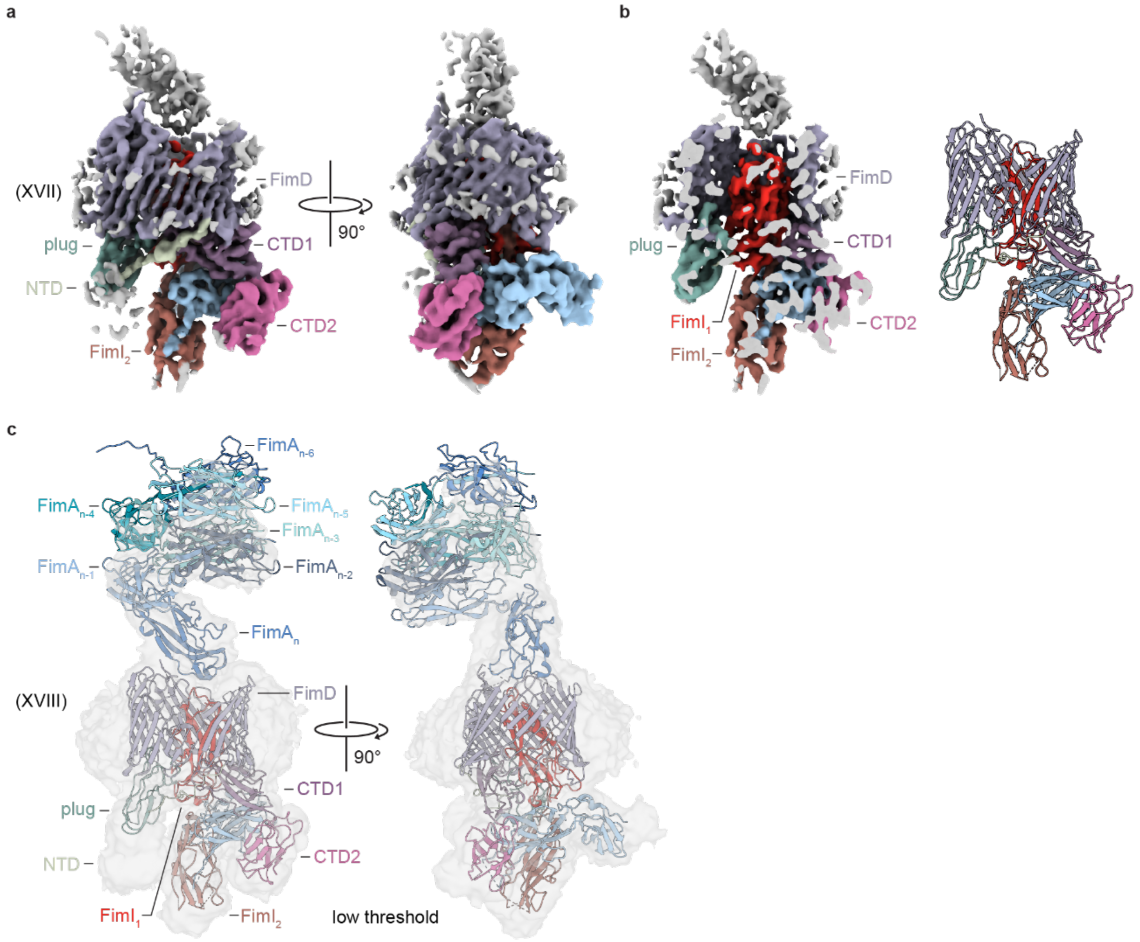

Supplementary Figure 15 | Incorporation of a second FimI subunit by the assembly platform FimD. a) Cryo-EM map (XVII) of the FimDHGFA$_n$I$_2$C$_{His}$ complex. **b)** Slice through the map in (a) so that both incorporated FimI subunits are visible (left) and cartoon representation of the model built into map XVII (right). **c)** Cryo-EM map (XVIII) of the FimDHGFA$_n$I$_2$C$_{His}$ complex in which the model from (b), PDB-ID 5OH0 and an additional FimA subunit from 5OH0 were rigid-body fitted using UCSF Chimera. The map is displayed at a low threshold so that the transition to the pilus rod is visible.

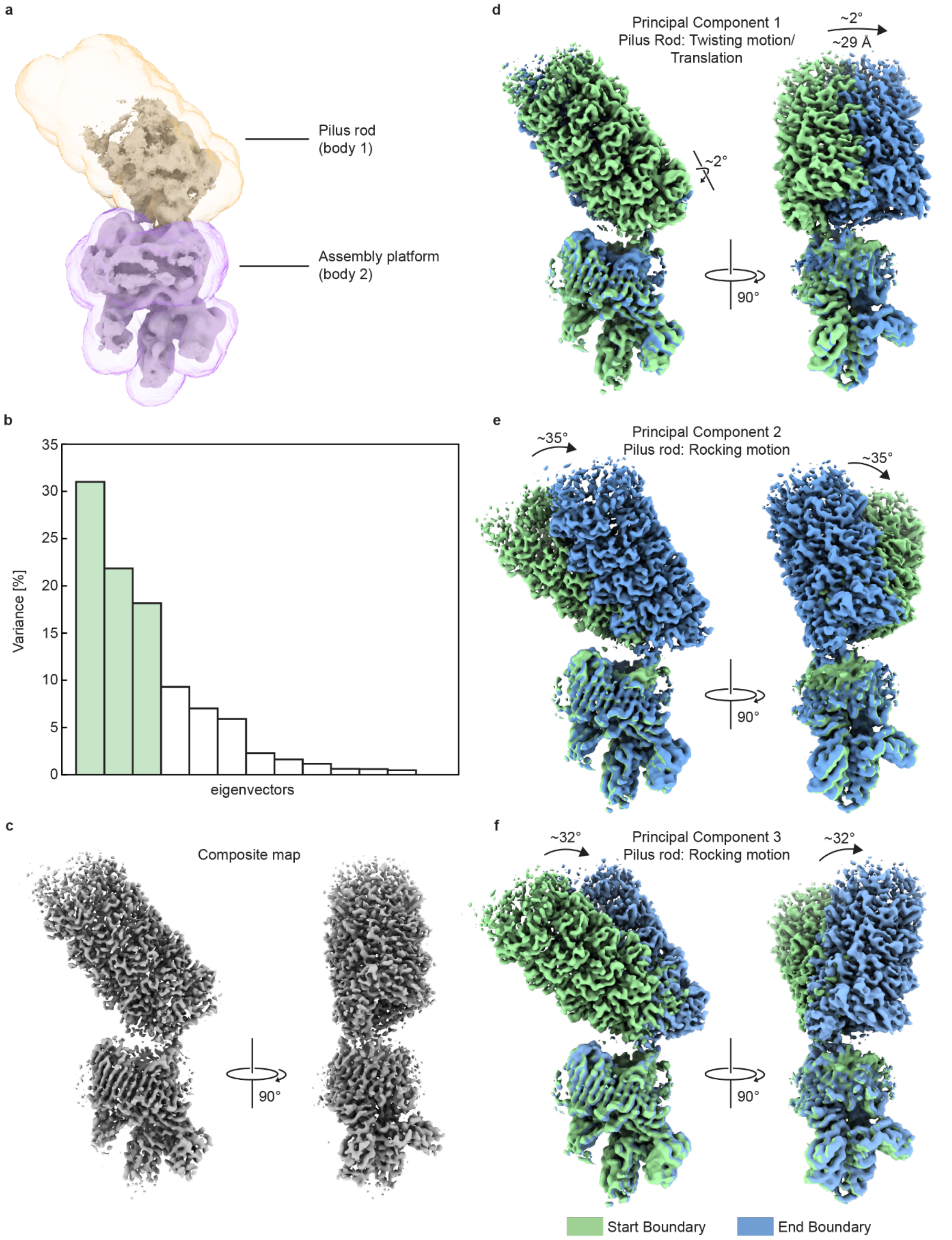

Supplementary Figure 16 | Analysis of angular flexibility of the pilus rod by multi-body refinement reveals distinct motions in the FimDHGFA$_n$IC$_{His}$ complex. a) Masks used for the two bodies in multi-body refinement. The mask surrounding the pilus rod (body 1) is colored orange while the mask for the FimI-bound assembly platform (body 2) is colored rose. **b)** Eigenvectors and their contribution to variance. The first three eigenvectors are colored green. Their corresponding motions are displayed in panels d) – f). **c)** Composite map of the two bodies from multi-body refinement. **d)** Principal component 1 shows a twisting/translation motion around the pilus rod axis. **e)** Principal component 2 shows a left to right rocking motion. **f)** Principal component 3 shows front to back rocking motion of the pilus rod.

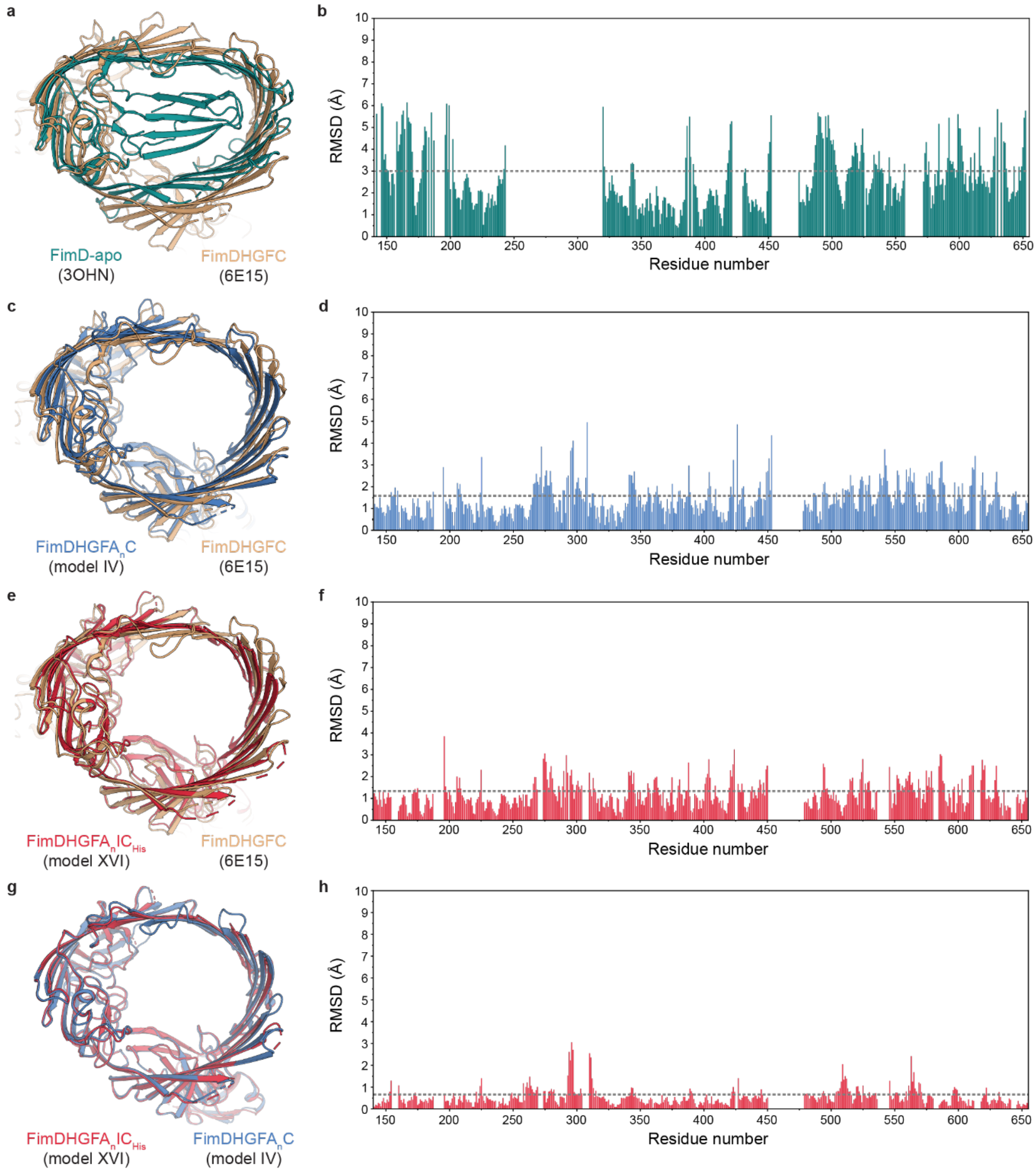

Supplementary Figure 17 | Pairwise structural alignment and plot of root mean square deviations (RMSD) of different FimD pore conformations. a) Structural alignment of FimD-apo (PDB-ID 3OHN) to conformer 2 of the FimDHGFC complex (PDB-ID 6E15) based on residues 140-665. **b)** Corresponding plot of the pairwise RMSD of C_α atoms of the two complexes. The overall RMSD of 3.00 Å is shown as a dashed line. **c)** Structural alignment of the $FimDHGFA_nC$ complex (model IV) with the FimDHGFC complex (PDB-ID 6E15). **d)** Corresponding plot of the pairwise RMSD of C_α atoms of the two complexes. The overall RMSD of 1.58 Å is shown as a dashed line. **e)** Structural alignment of the $FimDHGFA_nIC_{His}$ complex (model XVI) with the FimDHGFC complex (PDB-ID 6E15). **f)** Corresponding plot of the pairwise RMSD of C_α atoms of the two complexes. The overall RMSD of 1.34 Å is shown as a dashed line. **g)** Structural alignment of the $FimDHGFA_nIC_{His}$ complex (model XVI) with the $FimDHGFA_nC$ complex (model IV). **h)** Corresponding plot of the pairwise RMSD of C_α atoms of the two complexes. The overall RMSD of 0.66 Å is shown as a dashed line.

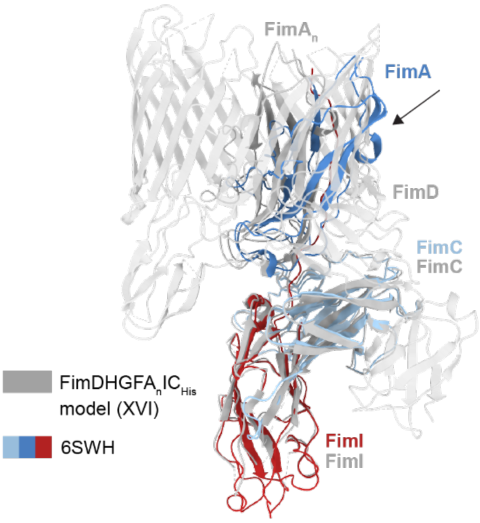

Supplementary Figure 18 | Alignment of the X-ray structure of the FimAIC$_{His}$ complex with the FimDHGFA$_n$IC$_{His}$ complex. The alignment was performed based on the chaperone FimC and involved chains D+E+F of 6SWH [6] and the FimDHGFA$_n$IC$_{His}$ complex (model XVI), RMSD= 0.69 Å. The FimAIC$_{His}$ complex is displayed in colors while the FimDHGFA$_n$IC$_{His}$ complex is displayed in grey. FimC and FimI of the FimAIC$_{His}$ complex align well with their respective counterparts in the FimDHGFA$_n$IC$_{His}$ complex. However, FimA has a different angle with respect to FimI and would clash with the β-barrel of the usher FimD in the FimDHGFA$_n$IC$_{His}$ complex (indicated with an arrow).

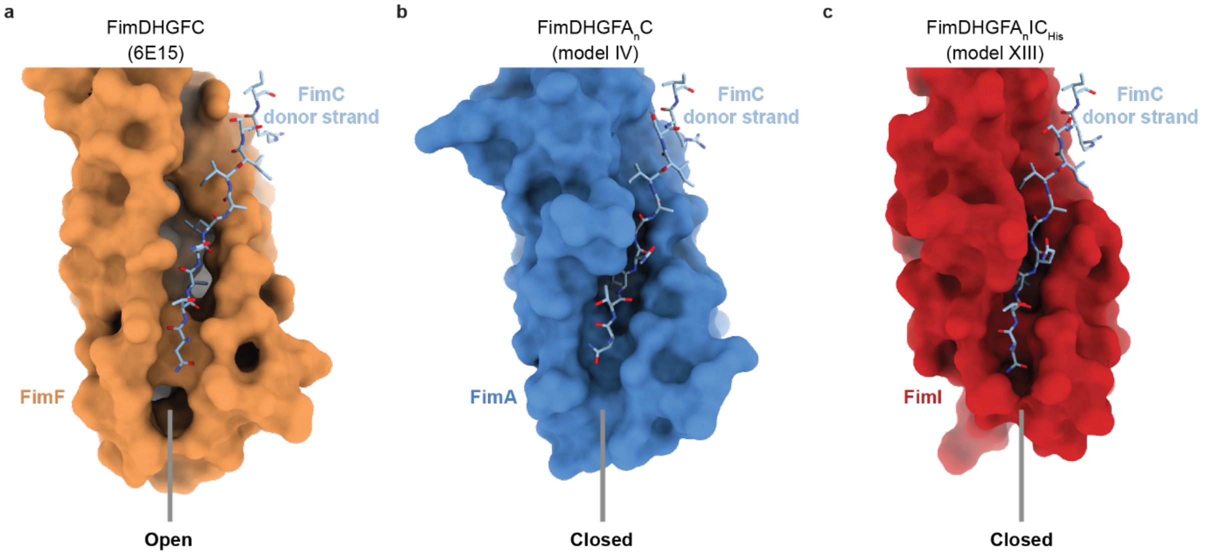

Supplementary Figure 19 | Comparison of P5 pockets of pilin-chaperone complexes bound on the periplasmic side of the usher FimD. a) Surface representation of FimF complemented by the G1 strand of FimC (shown as sticks) as part of the FimDHGFC complex (PDB-ID 6E15) [2]. The P5 pocket of the FimF subunit is in an open conformation. **b)** Surface representation of FimA complemented by the G1 strand of FimC (shown as sticks) as part of the FimDHGFA$_n$C complex (model IV). The P5 pocket of FimA is closed. **c)** Surface representation of FimI complemented by the G1 strand of FimC (shown as sticks) as part of the FimDHGFA$_n$IC$_{His}$ complex (model XIII). The P5 pocket of FimI is in a closed conformation.

Supplementary Table 1 | Cryo-EM data collection, refinement and validation statistics for complex FimDHGFA$_n$C.

	#1 Map I<br>Pilus rod and FimA-bound usher<br>(EMD-50755)	#2 Map II<br>FimDHGFA$_n$C<br>Pilus rod – Multibody 1<br>(EMD-50751)<br>(PDB 9FTT)	#3 Map III<br>FimDHGFA$_n$C<br>Usher – Multibody 2<br>(EMD-50828)<br>(PDB 9FW9)	#4 Map IV<br>FimDHGFA$_n$C<br>Local refinement 1<br>(EMD-50829)<br>(PDB 9FWB)	#5 Map V<br>FimDHGFA$_n$C<br>Local refinement 2<br>(EMD-50838)<br>(PDB 9FWZ)	#6 Map VI<br>FimD-tip conformer 1<br>(EMD-50861)<br>(PDB 9FY9)	#7 Map VII<br>FimD-tip conformer 2<br>(EMD-50954)	#8 Map VIII<br>Tip-to-rod-transition<br>(EMD-50839)<br>(PDB 9FX0)	#9 Map IX<br>Pilus tip and rod –<br>Conformer 1<br>(EMD-50773)	#10 Map X<br>Pilus tip and rod –<br>Conformer 2<br>(EMD-50796)	#11 Map XI<br>Pilus tip and rod –<br>Conformer 3<br>(EMD-50806)	#12 Map XII<br>Pilus tip and rod –<br>Conformer 4<br>(EMD-50809)
**Data collection and processing**												
Magnification	130,000x	130,000x	130,000x	130,000x	130,000x	130,000x	130,000x	130,000x	130,000x	130,000x	130,000x	130,000x
Voltage (kV)	300	300	300	300	300	300	300	300	300	300	300	300
Electron exposure (e–/Å^2)	64	64	64	64	64	64	64	64	64	64	64	64
Defocus range (μm)	-1.0 to -2.8	-1.0 to -2.8	-1.0 to -2.8	-1.0 to -2.8	-1.0 to -2.8	-1.0 to -2.8	-1.0 to -2.8	-1.0 to -2.8	-1.0 to -2.8	-1.0 to -2.8	-1.0 to -2.8	-1.0 to -2.8
Pixel size (Å)	0.65	0.65	0.65	0.65	0.65	0.65	0.65	0.65	0.65	0.65	0.65	0.65
Symmetry imposed	C1	C1	C1	C1	C1	C1	C1	C1	C1	C1	C1	C1
Initial particle images (no.)	10,241,804	10,241,804	10,241,804	10,241,804	10,241,804	10,241,804	10,241,804	10,241,804	10,241,804	10,241,804	10,241,804	10,241,804
Final particle images (no.)	119,055	119,055	119,055	119,055	119,055	242,477	98,487	127,989	21,787	21,085	11,770	33,742
Map resolution (Å)	4.1	3.6	3.9	3.5	3.6	3.3	4.2	3.1	3.9	4.0	4.4	3.9
FSC threshold	0.143	0.143	0.143	0.143	0.143	0.143	0.143	0.143	0.143	0.143	0.143	0.143
**Refinement**												
Initial model used (PDB code)		5OH0	6E15, 4DWH, Alphafold	6E15, 4DWH, Alphafold	6E15, 4DWH, Alphafold	6E15		5OH0, 3JWN				
Model resolution (Å)		3.7	4.0	3.6	3.8	3.4		3.1				
FSC threshold		0.5	0.5	0.5	0.5	0.5		0.5				
Map sharpening *B* factor (Å^2)		-89.8	-96.8	-84.9	-97.0	-91.4		-83.4				
Model composition												
Chains		10	4	4	5	5		7				
Non-hydrogen atoms		10062	8881	8881	9987	10974		7673				
Protein residues		1437	1172	1172	1331	1446		1088				
Ligands		--	--	--	--	--		--				
B factors (Å^2)												
Protein (min/max/mean)		51.90/104.66/65.41	32.72/116.99/62.58	4.66/91.83/34.88	22.60/114.42/58.49	0.00/80.90/16.99		9.47/152.23/37.31				
Ligand		--	--	--	--	--		--				
R.m.s. deviations												
Bond lengths (Å)		0.002	0.003	0.003	0.002	0.003		0.003				
Bond angles (°)		0.466	0.519	0.532	0.484	0.465		0.477				
Validation												
MolProbity score		1.28	1.51	1.65	1.63	1.36		1.24				
Clashscore		5.31	6.40	6.86	7.37	6.51		4.04				
Poor rotamers (%)		0.00	0.00	0.00	0.00	0.10		0.00				
Ramachandran plot												
Favored (%)		98.31	97.14	96.01	96.57	98.17		97.77				
Allowed (%)		1.69	2.86	3.99	3.43	1.83		2.23				
Disallowed (%)		0.00	0.00	0.00	0.00	0.00		0.00				

Supplementary Table 2 | Cryo-EM data collection, refinement and validation statistics for complex $FimDHGFA_nIC_{His}$.

	#13 Map XIII FimI-bound usher (EMD-50843) (PDB 9FX8)	#14 Map XIV Pilus rod and FimI-Bound usher (EMD-50810)	#15 Map XV $FimDHGFA_nIC_{His}$ Pilus rod - Multibody 1 (EMD-50846) (PDB 9FXA)	#16 Map XVI $FimDHGFA_nIC_{His}$ Usher - Multibody 2 (EMD-50847) (PDB 9FXB)	#17 Map XVII $FimDHGFA_nI_2C_{His}$ Local refinement (EMD-50853) (PDB 9FXS)	#18 Map XVIII $FimDHGFA_nI_2C_{His}$ Pilus rod and usher (EMD-50812)
**Data collection and processing**						
Magnification	165,000x	165,000x	165,000x	165,000x	165,000x	165,000x
Voltage (kV)	300	300	300	300	300	300
Electron exposure (e–/Å^2)	68	68	68	68	68	68
Defocus range (µm)	-0.8 to -2.4	-0.8 to -2.4	-0.8 to -2.4	-0.8 to -2.4	-0.8 to -2.4	-0.8 to -2.4
Pixel size (Å)	0.51	0.51	0.51	0.51	0.51	0.51
Symmetry imposed	C1	C1	C1	C1	C1	C1
Initial particle images (no.)	8,207,346	8,207,346	8,207,346	8,207,346	8,207,346	8,207,346
Final particle images (no.)	165,186	32,594	32,594	32,594	41,257	41,257
Map resolution (Å)	3.6	6.4	4.0	4.3	4.2	4.3
FSC threshold	0.143	0.143	0.143	0.143	0.143	0.143
**Refinement**						
Initial model used (PDB code)	6SWH, model #4		5OH0	Model #4, Model #11	Model #11	
Model resolution (Å)	3.9		4.2	4.4	4.3	
FSC threshold	0.5		0.5	0.5	0.5	
Map sharpening *B* factor (Å^2)	-110.1		-92.5	-121.0	-97.2	
Model composition						
Chains	3		10	4	4	
Non-hydrogen atoms	7739		10050	8363	8536	
Protein residues	1000		1435	1091	1106	
Ligands	--		--	--	--	
B factors (Å^2)						
Protein (min/max/mean)	31.86/157.64/74.01		9.47/74.24/22.75	2.43/109.54/21.81	76.72/205.86/125.70	
Ligand	--		--	--	--	
R.m.s. deviations						
Bond lengths (Å)	0.003		0.002	0.002	0.002	
Bond angles (°)	0.448		0.451	0.492	0.422	
Validation						
MolProbity score	1.51		1.26	1.31	1.36	
Clashscore	4.85		4.96	5.70	6.53	
Poor rotamers (%)	0.00		0.00	0.00	0.00	
Ramachandran plot						
Favored (%)	96.19		98.02	98.10	98.60	
Allowed (%)	3.81		1.98	1.90	1.40	
Disallowed (%)	0.00		0.00	0.00	0.00	

References

1. Lomize, A.L., S.C. Todd, and I.D. Pogozheva, *Spatial arrangement of proteins in planar and curved membranes by PPM 3.0.* Protein Sci, 2022. **31**(1): p. 209-220.
2. Du, M., et al., *Handover mechanism of the growing pilus by the bacterial outer-membrane usher FimD.* Nature, 2018. **562**(7727): p. 444-447.
3. Sievers, F., et al., *Fast, scalable generation of high-quality protein multiple sequence alignments using Clustal Omega.* Mol Syst Biol, 2011. **7**(1): p. 539.
4. Gabler, F., et al., *Protein sequence analysis using the MPI Bioinformatics Toolkit.* Curr Protoc Bioinformatics, 2020. **72**(1): p. e108.
5. Zimmermann, L., et al., *A completely reimplemented MPI Bioinformatics Toolkit with a new HHpred server at its core.* J Mol Biol, 2018. **430**(15): p. 2237-2243.
6. Giese, C., et al., *Stochastic chain termination in bacterial pilus assembly.* Nat Commun, 2023. **14**(1): p. 7718.
